# How attachment affects users’ continued use intention of tourism mobile platform: A user experience perspective

**DOI:** 10.3389/fpsyg.2022.995384

**Published:** 2022-08-15

**Authors:** Guopeng Xiang, Qian Chen, Qiucheng Li

**Affiliations:** School of Tourism and Urban-Rural Planning, Zhejiang Gongshang University, Hangzhou, China

**Keywords:** tourism mobile platform, attachment theory, continued use intention, user experience, SOR framework

## Abstract

Continued use intention of customers is a critical factor in the development of tourism mobile platforms (TMP), which reflects the degree of users’ attachment to the platforms. However, existing research in this field intends to investigate users’ attachment to a TMP by focusing on the overall cognitive satisfaction of the users, which deviates from the “cognition-affect” framework in psychology. Following the stimulus–organism–response (S-O-R) framework, this paper draws upon the attachment theory and the user experience theory, and proposes a model depicting how service experience of TMP affects users’ intention to keep using the TMP through the mediation effect of platform attachment. The empirical results (N = 276) showed that functional experience and social experience positively affect TMP users’ development of platform attachment (i.e., platform dependence and platform identity), which in turn enhance their intention to continuously obtain and provide tourism information *via* the TMP. This study expands the research on the continued use of TMP from an attachment perspective and contributes to the field in both theoretical and practical levels.

## Introduction

The promotion and popularity of smartphones has contributed to the rapid growth of the tourism mobile platform (TMP) market (Choi et al., 2019). According to the statistical report of China Internet Network Information Center, June 2021, the number of online travel booking users in China had reached 367 million, an increase of 24.11 million over the end of 2020, accounting for 36.3% of the global internet users. This indicates that more and more consumers plan and make decisions through TMPs. Meanwhile, with the increasing competition in the industry, various application platforms such as reservation, strategy, and social networking have developed rapidly in a short period of time (Palos-sanchez et al., 2021), providing TMP users with diverse options. Research has shown that the cost of acquiring a new customer is five times that of retaining an old customer (Schefter and Reichheld, 2000; Bhattacherjee, 2001; Hill and Alexander, 2006). In order to gain sustainable competitive advantage, TMP operators should not only try to attract new users, but also need to retain old users. Therefore, how to retain and cultivate the sustainable adoption of users has become a critical issue for TMP companies and operators.

Previous studies on TMP have mainly focused on the adoption decision of users; comparatively less attention has been paid to the users’ post-adoption behavior (Ukpabi and Karjaluoto, 2017; Law et al., 2018). Although some scholars have addressed the importance of TMP users’ continued use the platform, they have mainly resorted to the expectation-confirmation logic, and predicted users’ continued use intention by evaluating their satisfaction with the TMP (Pan et al., 2016; Choi et al., 2019; Wu et al., 2021). This kind of satisfaction based on functional experience (e.g., perceived usefulness and perceived ease of use) deviates from the “cognitive-affect” relationship in psychology. In fact, continued use intention is largely an irrational matter with emotional factors playing an important role therein (Weiss and Cropanzano, 1996; Zhao et al., 2012). With the popularity and promotion of TMP in tourism and daily life, people can not only improve tourism efficiency through functional services provided by TMPs (e.g., searching accommodation, tourism activities, flight and reservation information; Lu et al., 2015; Schaefer, 2016), but also interact and share with other users on tourism related information. This interactional and altruistic experience provided by TMPs will promote the development of emotional ties between users (Li et al., 2021), which would translates into users’ attachment to the platform, i.e., platform attachment. Platform attachment, consisting of the two dimensions of platform dependence and platform identity, have been argued to have significant implications for users’ behaviors (Li et al., 2021), particularly in driving users to maintain or strengthen relationships with the platform (Park et al., 2006), and developing the intention to keeping using the platform.

Based on the above discussion and in line with the stimulus–organism–response (SOR) framework, we use user experience (functional experience, social experience and altruistic experience) as stimulus variables (S), platform attachment as organism variables (O), and continued use intention as the response variable (R), to explore the influence mechanism of user experience on TMP users’ continued use intention. This paper integrates the user experience theory and the attachment theory, and investigates how the uses’ functional, social, and altruistic experience in using a TMP to affect their intention to continue using the TMP through the development of platform dependence and platform identity. This combination of user experience theory and attachment theory helps to enhance our understanding of the mechanism underlying users’ TMP continuous using decision. Empirical findings of the paper are also helpful for TMP operators to formulate effective relationship marketing strategies and improve market competitiveness of the TMP. As such, this paper contributes to the literature in both theoretical and practical aspects.

## Literature review

### Stimulus-organism-response model

The stimulus-organism-response (SOR) model was first proposed by Mehrabian and Russell (1974) to describe the process of individual behavioral response caused by external environmental stimuli. The framework indicates that when individuals are aware of the existence of stimulus sources, they will form cognitive and emotional reactions, which then lead to approaching or avoiding behavioral responses (Zhang and Xu, 2019). Prior studies have applied SOR framework in the field of e-commerce live streaming (Xu et al., 2020) and health care (Chen and Yin, 2021), and rarely in the field of TMP. This study applies creatively the tenet of the SOR framework to the context of tourists’ use of TMPs. A TMP is an operator that combines the internet and tourism resources and provides comprehensive services for tourists, promoting the transfer of user groups to intelligent terminals. It is the “stimulus” factor of the external environment that causes changes in users’ consumption behaviors. Drawing upon the user experience theory, we propose user experience is response of an individual that short-term stimulation or long-term experience (Schmitt, 1999; Lemon and Verhoef, 2016), thus it essentially related to external environmental stimuli (Chen, 2021). This study uses the concept of user experience to capture TMP as an external environmental stimulus. In the SOR framework, O (organism) refers to the cognition and emotion of the organism, such as emotional and cognitive state of an individual (Xie et al., 2019). TMP attachment in this study refers to the user’s perceived cognitive and emotional connectedness with the product or service provided by the platform, thus corresponding to the O element of the SOR framework. R (response) refers to individuals’ response to the cognitive and emotional changes. As thus, this study considers the users’ continued use intention of TMP representing the R element.

In sum, based on the SOR framework, the research model of the study is established by taking user experience as the stimulus (S), platform attachment as the cognitive and emotional consequences (O), and continued use intention as the response (R).

### User experience

User experience is an individual’s response to stimulation and a subjective psychological emotion generated by people after short-term stimulation or long-term experience (Schmitt, 1999; Lemon and Verhoef, 2016). In the field of interaction design, Forlizzi and Battarbee (2004) found that experience originates from the interaction process between individuals and products or services, mainly including the functions of products or services, the psychological feelings and usability of the use process. Nambisan and Watt (2011) also proposed that online community experience is the all-round experience obtained by users in the process of online community interaction, and indicated that community experience includes practical experience, hedonic experience, and usable experience.

Some prior researches have adopted user experience theory to explore online consumer behavior. For example, Huang et al. (2015) explored the impact of consumer experience on brand community loyalty. They found that information experience and entertainment experience significantly affect brand loyalty through the mediating role of community identity. Pang and Yang (2021) found the interactive experience of virtual brand community promotes customer citizenship behavior, which in turn recommends more people to join the community, and improves purchase intention. Kim et al. (2016) reported the effect of social experience on social network website loyalty, in which user attachment plays a mediating role. Notably, Lei and Zheng (2021) argued that the emotional and altruistic dimensions of tourist experience value are pivotal antecedents to customers’ continued use intention. Tourists’ continued use intention of TMP depends on the possibility and value perception of the potential consequences of the actual use or service experience (assessment) of tourists (Choi et al., 2021). Functional experience, social experience, and altruistic experience are important experience or perception components (Sweeney and Soutar, 2001; Yun et al., 2021). Thus, in the context of TMP, this paper adopts a three-dimensional frame of user experience (i.e., functional experience, social experience, and altruistic experience) to fuel the investigation of the formation of users’ continued use intention of a TMP.

### Attachment theory

Previous research has defined attachment as the result of continuous satisfaction of individual needs, which will form a behavior control system and unique behavior pattern dominated by the emotional connectedness developed between the individual and the object (Bowlby, 1982; Trairatvorakul, 2016). Thus, attachment can generate motivation and behavior (Aron et al., 2004; Park et al., 2010; Wan et al., 2017). If the object of attachment continues to meet individual needs (Zhao et al., 2012), individuals will pay extra time, energy, money, and other resources to the object of attachment to maintain a close relationship with it (Fedorikhin et al., 2008; Zhao et al., 2012; Choi, 2013).

Choi (2013) considered that the degree of the users’ attachment to information system (IS) products or services has an important impact on the users’ intention to engage in community behaviors. Indeed, it will take extra time and energy to participate in the community than to recommend to other users (Krishnamurthy, 2009). Therefore, attachment is an important parameter regarding users’ continuous participation and retention. This study contends that in the context of TMP, attachment is even more effective than loyalty and satisfaction in explaining users’ continued use intentions.

In the field of web site attachment research, retail websites become the object of individual attachment (Ren et al., 2012). That is, consumers connect the products or services of retail web sites with their own needs (Yan and Da-hai, 2008). The concept of platform attachment is derived from the concept of website attachment, and refers to the emotional connection of users developed with the platform (Kim et al., 2016). In the context of TMP, if a user becomes attached to the platform, he or she will keep using the products or services provided by the platform for their functional needs. For example, users can rely on a TMP’s reservation, transaction, and information search services before and during their travel to facilitate quality and efficiency of their travel (Okazaki and Mendez, 2013; Palos-sanchez et al., 2021).These services are associated with external and self-directed values and the users’ functional dependence on the TMP (Setterstorm et al., 2013; Zhang et al., 2019). As such, we define this functional attachment as platform dependence. In addition, TMP users can also communicate with each other and share high-quality tourism information with other users on the platform (Ren et al., 2012; Lai, 2015). This would form emotional connections between users and further enhance the users’ recognition of the value of the platform (Yun et al., 2021). This social and emotional dimension of platform attachment is usually referred to as platform identity.

Attachment has been considered an important driver of users’ continued use intention. When users develop dependency on and identity with an object, they are inclined to maintain and strengthen their relationship with the object (Aron et al., 2004). As such, the products or services provided by TMP should ensure that they are attractive enough to retain users. This study thus adopts the attachment theory, as discussed above, to explain continued use intention in the context of TMP.

### Continued use intention

In previous studies, the discussion of intention to continue using the IS mainly focused on the general IS field (Bhattacherjee, 2001; Limayem et al., 2007), which mainly means that users continue to use the system after the initial acceptance of new technology. With the development of mobile network technology, the meaning of continued use intention has been explored in more detail in different network environments. It not only refers to the uses’ intention to continue obtaining information and purchasing products or services (Wei et al., 2021; Cheng and Shao, 2022), but also highlights the uses’ intention to continue providing information about their needs in community or TMP (Tang et al., 2022). In the big data context, users can continuously obtain a large amount of useful recommendation information through tourism platforms, websites, and communities (Huang et al., 2015) to make tourism or purchase decisions. In the field of e-commerce, continued use intention was defined as the customers’ intention to revisit the website or consider purchasing goods or services from the website in the future (Flavián et al., 2006; Cyr et al., 2009). For users who are willing to use TMP continuously, they are not only willing to search for tourism information during travel, but also willing to spend time on generating content and sharing knowledge after travel (Cyr et al., 2009; Molinillo et al., 2018). For example, Krishnamurthy (2009) proposed that the community’s intention to continue to participate include the users’ inclination to spend more time and energy to answer other users’ questions, provide suggestions related to functions, and develop user-driven marketing activities.

Some researchers have also explored the influential factors of continued use intention. Hsiao et al. (2015) explored the influencing factors of the intention to continue using mobile social applications from the perspective of satisfaction, customer value and habits, and verified that users’ satisfaction and habits are important antecedents of their continued use of mobile social applications. Pan et al. (2016) indicated that satisfaction, perceived usefulness, and service quality are the critical driving factors of users’ intention to continue using tourism APPs. Choi et al. (2019) found that satisfaction, trust, and familiarity significantly affect tourists’ intention to continue using the tourism APP. Choi (2013) showed that attachment is an important antecedent of users’ intention to participate in the community in the context of IS. Meanwhile, some researchers found emotional trust and perceived emotional value also affect consumer loyalty in the context of live e-commerce, which ultimately influence the users’ purchase intention (Zhou and Tong, 2022). For instance, Castañeda et al. (2019) explored users’ loyalty to tourism APPs and their continued use behavior from the perspective of usefulness and perceived value (Oliver, 1980).

### Research model and hypotheses

**Figure 1:**
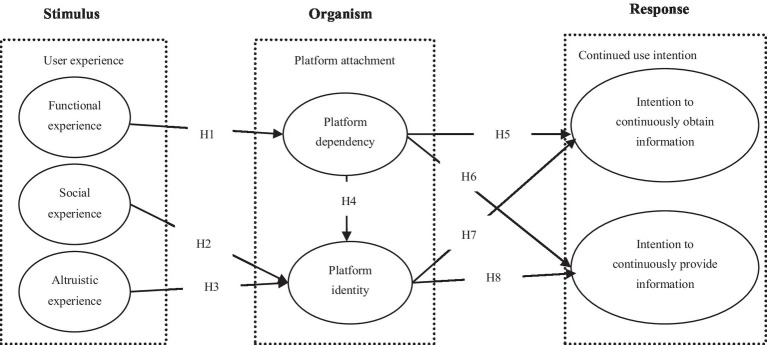
Conceptual model of the study.

Based on the SOR framework, we combine attachment theory and user experience to explore the driving factors of TMP users’ continued use intention. Following Bhattacherjee (2001), Chen (2007) and Ye et al. (2021), we propose that consumers in social media have strong autonomy. That is, consumers are active content creators rather than merely passive recipients of information (Peters et al., 2013), and TMPs are an important platform for obtaining, disseminating, and exchanging tourism-related information (Chen, 2021). Therefore, we use two constructs to operationalize users’ intention to continuing using (continuously obtain tourism information and continuously provide tourism information). The research model is presented in Figure 1.

### User experience and platform attachment

Functional experience refers to the perceived usefulness of using a IS to improve its performance level (Davis, 1989). It is oriented by target behavior, reflecting that an important purpose of people using the IS to obtain valuable information and help (Qu and Zhang, 2015). Existing research has shown that IS products and services can induce self-efficacy of the users and eventually lead to attachment (Choi, 2013). For instance, Park et al. (2006) found that brand performance attributes can create a kind of self-efficacy that enables consumers to effectively complete goals and tasks, and they will develop a strong sense of attachment. Similarly, Zhang et al. (2019) discovered the recommendation system such as push popular content based on users’ browsing history and preferences of short-form video application can enhance users’ attachment to short-form video application. Choi et al. (2019) also pointed out that when the travel app provides reliable price information and satisfactory transaction functions, the users will develop a sense of attachment and believe that they can rely on the app when traveling.

Based on the above discussion, we predict that if the products or services of a TMP can timely meet users’ travel needs, the users will feel an enhanced self-efficacy by using the TMP, and thereby develop a functional dependence on the TMP (Wan et al., 2017). This leads to the following hypothesis:

*H1*: Functional experience with a TMP positively influences the users’ platform dependence.

Social experience refers to the experience that consumers get by communicating with other members in the community (Huang et al., 2015). Consumers can interact with community members with similar interests and establish a close relationship with them (Bhattacharya and Sen, 2003; Ashforth et al., 2008). This will form emotional attachment between the members. For example, Achen (2019) observed that high-quality interactions can establish a solid emotional connection between users, which will enhance their attachment to each other (Fiedler and Sarstedt, 2014). According to the theory of emotional transfer, emotional attachment developed in interpersonal relationships can be transferred to platform attachment (Li et al., 2021). In view of this, this paper proposes that the interactions between users with similar value claims on TMP can enhance their perceived connectedness with each other (Thomson, 2006), which will translate into their recognition and identity with the platform *per se*. Based on the above discussion, the following hypothesis is proposed:

*H2*: Social experience with a TMP positively influences the users’ platform identity.

Altruistic experience refers to the users’ perception that their information provision or sharing behavior has contributed to other users’ convenience and benefits (Butler et al., 2002; Morris, 2006; Zheng et al., 2013). In this respect, Yun et al. (2021) reported the positive impact of users’ perceived altruistic value on community identity in the context of online encyclopedia. In the context of knowledge sharing virtual community, users provide knowledge contribution through the community platform to help others solve problems (Liao et al., 2013), and gain a sense of pleasure (Wasko and Faraj, 2005). This will naturally promote the users’ recognition of the platform and develop a sense of identity with it. We believe that the information of the real travel and consumption experience of consumer is published in TMP, which meets his or her altruistic needs and strengthens their self-concept connection with platform enterprises. It is not only the embodiment of self-worth, but also the recognition of the platform value concept. Therefore, we put forward the following hypothesis:

*H3*: Altruistic experience with a TMP positively influences the users’ platform identity.

### Platform dependence and platform identity

Platform dependence and platform identity have different psychological properties (Kyle et al., 2004). The attribute of platform dependence reflects the utilitarian value of the platform and is an external benefit (Setterstorm et al., 2013). By contrast, the platform identity attribute reflects the emotional connection between users and recognition of platform values (Yun et al., 2021). When users perceive the benefits and values provided by the platform, they will recognize the platform (Dholakia et al., 2004). The maintenance of emotional connection is actually built upon external effective value. Thus, we propose the following hypothesis:

*H4*: Platform dependence positively influences platform identity.

### Platform attachment and continued use intention

Platform attachment refers to the emotional bond developed between the users and the platform (Kim et al., 2016). As have discussed earlier, we maintain that platform attachment can be deconstructed into two dimensions, namely platform dependence and platform identity. Platform attachment is an important parameter as to the connection between users and platforms. Functional dependency has been defined as how well a setting and condition support the user’s goal achievement (Jackson et al., 1996; Trauer and Ryan, 2005), which will in turn influence the user’s emotional states and behavior (Wu, 2015). For example, existing research has observed that users who rely on a TMP will continuously search for tourism services and information through the platform during their travel or at the destination (Höpken et al., 2010). Chen et al. (2014) and Beck et al. (2014) found that the technical support provided by social media promotes the users to share knowledge on the platforms. Similarly, Wan et al. (2017) stressed that users’ functional dependence on a social media affects their intention of content creation therein.

In the field of online virtual community, some researchers have explored a series of behaviors caused by website attachment or community identity. For example, Jiang and Dong (2008) found that consumers’ attachment to retail websites positively affects their browsing and shopping loyalty to the websites. Also, Ren et al. (2012) indicated that members’ attachment to an online community can improve the members’ participation as well as retention of members on the website. In the context of knowledge sharing, Ma and Agarwal (2007) indicated that recognition of an online community implies the users’ efforts to express and show to others, which thereby promotes their knowledge contribution behavior. Yun et al. (2021) also found that community identity promotes the intention of online encyclopedia users to contribute continuously to the free content. Likewise, Yang and Lai (2010) found that the sense of community belonging is a critical antecedent of the users’ intention to continuously provide information.

Considering that continuously obtaining and providing tourism information is an important behavior to maintain the relationship between users and TMPs, we build upon the existing relevant studies and propose the following hypotheses:

*H*5: platform dependence positively influence the users’ intention to continuously obtain information from the TMP

*H*6: platform dependence positively influence the users’ intention to continuously provide information on the TMP

*H*7: platform identity positively influence the users’ intention to continuously obtain information from the TMP

*H*8: platform identity positively influence the users’ intention to continuously provide information on the TMP

## Methology

### Measurement

The questionnaire was designed by taking following steps. Firstly, an initial questionnaire is developed after a thorough review of the existing relevant literature. Secondly, the measurement scales were refined and wording of some items were adjusted after a round of expert review of the initial questionnaire. Thirdly, a pilot test (N = 176) was conducted in a university in Hangzhou, China to preliminary assess reliability and validity of the measurement. This three-step process led to the final questionnaire for the survey of the study. A Likert-type 5 point scale is used to access each of the measurement items with 1 representing “strongly disagree” and 5 standing for “strongly agree.” The measurement scales are presented in Table 1.

**Table 1:** Measurements of the constructs.

Construct	Items	Sources
Functional experience (FE)	FE1: Reliable information of tourist routes and scenic spots	Csikszentmihalyi and Csikszentmihalyi (1989)
	FE2: Convenient and reliable reservation of tourism products	
	FE3: Like the pushed content and update it timely	
	FE4: Perfect payment function and transaction security	
	FE5: During the use period, the account personal information is guaranteed	
Social experience (SE)	SE1: I can have very good communication with people who are also interested in traveling	Money (2004), Sha et al. (2010), Qu and Zhang (2015)
	SE2: This platform can communicate with others around their favorite tourist destinations	
	SE3: This platform can make me meet new friends who have the same hobby of traveling	
Altruistic experience (AE)	AE1: Sharing travel information on this platform can help others	Butler et al. (2002), Morris (2006), Yun et al. (2021)
	AE2: Sharing travel strategies and writing travel notes through this platform can help other friends who like to travel	
	AE3: Online hotel or scenic spot evaluation through this platform	
Platform dependency (PD)	PD1: The platform can meet the actual needs of our travel process	Tsai (2011), Jap and Anderson (2007)
	PD2: This platform can provide professional services and help for my travel	
	PD3: This platform solves the specific problems encountered in the tourism process for me	
Platform identity (PI)	PI1: I feel that this platform is a part of my travel life	Li et al. (2021)
	PI2: This platform records my travel experience	
	PI3: This platform reflects my values	
	PI4: I have a strong identification with this platform	
Intention to continuously obtain information (ICO)	ICO1: I will continue to use the tourism platform to obtain tourism information	Bhattacherjee (2001), Zhang (2010), Chen (2007)
	ICO2: I will continue to browse tourism related information on this platform	
	ICO3: I will continue to seek help on this platform	
Intention to continuously provide information (ICP)	ICP1: I will continue to share my travel experience on this platform	Bhattacherjee (2001), Zhang (2010), Chen (2007)
	ICP2: I will continue to answer questions from other users seeking travel help	
	ICP3: I will continue to share my travel knowledge on this platform in an effective way	

### Data collection

Our respondents are users of Ctrip for more than 1 year. Ctrip is a leading TMP in China. In 2021, the number of Ctrip users reached 220 million. We collect data both online and offline. The online survey was mainly conducted through “wjx.cn” (a charge online survey website of China). As for the offline survey, the questionnaires were randomly distributed in the West Lake Scenic Area in Hangzhou, China, which is the main gathering place for visitors to Hangzhou. When approaching a respondent, we first asked whether he or she had used Ctrip, and only respondents who had relevant experience of the TMP were invited to participate in the survey. The survey was conducted in May, 2022, and 310 completed questionnaires were collected. After eliminating questionnaires with missing date and obviously consistent answers, 276 valid questionnaires were obtained and used in subsequent analyses, resulting in an effective response rate of 89%. Loehlin (1992) and Mueller (1997) suggested that the sample size for structural equation model (SEM) is at least 100, and in most cases a sample with more than 200 cases is better (Kline, 2011). Therefore, we feel that a sample size of 276 for the study was generally acceptable to generate reliable results.

The sample consists of relatively more females (56.7%) than males (43.3%).Over 75% of the participants are within the age between 25 and 34. As to education background, the sample is characterized by a rather high education level with 89.9% of the participants having a bachelor degree or above. The sample demographics are consistent with the previous research that has reported a well-educated young groups who use TMP frequently for their travel plans (Pan et al., 2016; Choi et al., 2019).

### Data analysis

This study mainly used SEM to test the conceptual model and research hypothesis. The internal consistency of the scale was measured by the Cronbach’s *α* coefficient in SPSS21.0. On this basis, we further analyze the data in two steps. Firstly, the reliability and validity of the variable measurement are verified by confirmatory factor analysis (CFA), and then the structural model was tested to evaluate the proposed theoretical model and hypotheses therein. The data analysis of this study was carried out in Amos 21.0.

## Results

### Data assessment

To test the potential risk of common method variance (CMV), Harman single factor analysis was performed (Podsakoff and Organ, 1986). The results showed that there was no single factor that accounted for more than 50% of the total variance, so common method variance was not serious problem in present study.

### Reliability and validity test

Firstly, we measured Cronbach’s α coefficient for assessing internal consistency. All α values exceeded 0.75, which means high reliability levels. Secondly, a confirmatory factor analysis was conducted to ensure convergent validity of the constructs. As shown in Table 2. The composite reliability (CR) values of all constructs exceeded 0.65; the average variance extracted (AVE) values exceeded 0.5 and the standard factor loadings exceeded 0.65. These results indicate good convergent validity (Fornell and Larcker, 1981). Finally, we measured the square root of AVE values of all variables and correlation coefficients between variables to test the discriminant validity. The square root of the AVE values of each variable (bold values in Table 3) is higher than the correlation coefficients of other variables, which means good discriminant validity (Sitgreaves, 1979).

**Table 2:** Confirmatory factor analysis results.

Construct	Item	Parameter significance estimation				Standard loading	SMC	CR	AVE	Cronbach’s α
		Unstd.	S.E.	*t*-value	*P*-value					
FE	FE1	1				0.831	0.691	0.873	0.581	
	FE2	0.930	0.066	14.13	***	0.783	0.613			
	FE3	0.728	0.063	11.589	***	0.668	0.446			0.872
	FE4	0.875	0.062	14.003	***	0.778	0.605			
	FE5	0.848	0.064	13.156	***	0.740	0.548			
SE	SE1	1.000				0.777	0.604	0.790	0.557	
	SE2	0.991	0.101	9.812	***	0.741	0.549			
	SE3	0.967	0.099	9.749	***	0.720	0.518			0.789
AE	AE1	1.000				0.787	0.619	0.845	0.645	
	AE2	1.149	0.090	12.802	***	0.869	0.755			0.843
	AE3	0.966	0.079	12.177	***	0.749	0.561			
PD	PD1	1.000				0.775	0.601	0.811	0.588	
	PD2	1.009	0.093	10.857	***	0.811	0.658			0.809
	PD3	0.882	0.084	10.519	***	0.712	0.507			
PI	PI1	1.000				0.811	0.658	0.824	0.542	
	PI2	0.871	0.081	10.741	***	0.675	0.456			
	PI3	0.835	0.081	10.348	***	0.651	0.424			0.821
	PI4	0.971	0.079	12.317	***	0.793	0.629			
ICO	ICO1	1.000				0.824	0.679	0.832	0.624	
	ICO2	0.999	0.083	12.084	***	0.796	0.634			0.831
	ICO3	0.972	0.083	11.735	***	0.747	0.558			
ICP	ICP1	1.000				0.845	0.714	0.684	0.867	
	ICP2	0.842	0.060	13.936	***	0.786	0.618			0.865
	ICP3	1.002	0.068	14.684	***	0.849	0.721			

**Table 3:** Square root and correlation coefficient of mean extracted variance.

	AVE	SE	PI	PD	FE	ICP	ICO	AE
SE	0.557	0.746						
PI	0.542	0.710	0.736					
PD	0.588	0.481	0.629	0.767				
FE	0.581	0.632	0.669	0.518	0.762			
ICP	0.867	0.630	0.642	0.578	0.544	0.931		
ICO	0.624	0.662	0.653	0.607	0.596	0.592	0.790	
AE	0.645	0.527	0.477	0.478	0.474	0.560	0.601	0.803

### Structural model test

**Figure 2:**
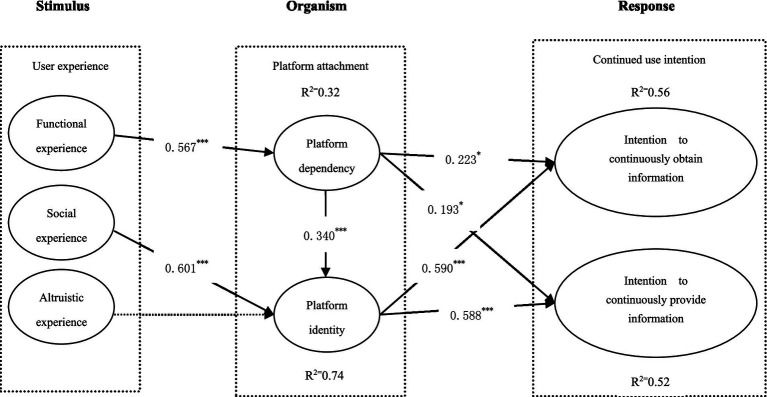
Structural model results. ***p < 0.001, *p < 0.05.

We evaluated the theoretical model and hypotheses by using AMOS 21.0, the results show a good model fitness: *χ*^2^/df = 1.574, AGFI = 0.873, CFI = 0.959, GFI = 0.898, NFI = 0.896, RMSEA = 0.046. All the fitting indices of the model reached the recommended value, which indicates a good fitness (Bentler, 1983). As shown in Figure 2. The results prove that functional experience has a positive impact on platform dependency (*β* = 0.567，*p* < 0.001), which positively influenced platform identity (*β* = 0.340, *p* < 0.001), intention to continuously obtain tourism information (*β* = 0.223, *p* < 0.05), and intention to continuously provide tourism information (*β* = 0.193, *p* < 0.05).Social experience has a significant impact on platform identity (*β* = 0.601, *p* < 0.001)，which positively influenced intention to continuously obtain tourism information (*β* = 0.590, *p* < 0.001), and intention to continuously provide tourism information (*β* = 0.588, *p* < 0.001). Hypothesis 3 was not supported.

## Discussion and conclusion

### Summary of the results

This study applied the SOR framework to the TMP context, and systematically examined the impact of user experience on continued use intention *via* the mediation of platform attachment. An empirical research with a Ctrip user sample (*N* = 276) supported most of the proposed hypotheses, and some important conclusions are be drawn.

Firstly, we empirically investigated how user experience factors influence platform attachment and thereby trigger users’ intention to continue using the TMP, and some interesting findings were gained. Specifically, functional experience has a significant and positive impact on platform dependency, while social experience has a significant and positive impact on platform identity. Previous studies on TMP users’ continued use intention have mainly focused on instrumental attributes of the platform with little attention being directed to social and altruistic aspects of the platforms. This study revealed that functional experience and social experience with the TMP can positively affect the users’ attachment to the platform. More specifically, by using a TMP, the users can access travel information, make itinerary, conduct online booking and payment, which can effectively meet the users’ travel goals, thus forming platform dependence. Also, the users can disseminate and exchange tourism related information with other users, which could improve their social feelings such as self-fulfillment and self-actualization, and lead to platform identity. Surprisingly, altruistic experience with the TMP failed to exert any significant impact on the users’ platform identity. One possible reason for this result may be that the TMP users in our sample contribute to the platform with tourism-related knowledge and information mainly for utilitarian purposes, rather than identification with the platform.

Secondly, we decomposed platform attachment into platform dependence and platform identity, and empirically tested their impact on TMP users’ continued use intention. Previous studies have mainly discussed the direct relationship, such as perceived usefulness, perceived ease of use and satisfaction, between users’ intention to continue using and antecedents (Pan et al., 2016; Zhang et al., 2017; Choi et al., 2019). Meanwhile, the continued use intention also focused on the aggregation construct of uni-dimensional structure, and the two behaviors (e.g., intention to continuously obtain tourism information and intention to continuously provide tourism information) were not distinguished. Unlike prior studies, we further discussed the impact of platform dependence and platform identity on users’ intention to continue using a TMP. In addition, it also subdivides users’ intention to continue using, namely, intention to continuously obtain tourism information and intention to continuously provide tourism information. As such, this study serves as a meaningful starting point and provides avenues for future studies that aim to further probe into the mechanism underlying the users’ intention to continue using TMPs.

Thirdly, we found that platform identity has a greater impact on users’ intention to continue using the TMP than platform dependence. This may due to the fact that tourism activities are transient and infrequent in nature (Pan et al., 2016). Individuals who rely on a TMP usually use its products or services provided merely for tourism-related reasons. Therefore, the user usually has a relatively lower degree of attachment to a TMP. That is, different attachment levels have different effects on user behavior levels (Park et al., 2006). Intensity attachment will urge individuals to make sacrifices and inputs to maintain the sustainability of the relationship (Van Lange et al., 1997). For example, users satisfy themselves in their continuous interaction with other users, resulting in a sense of belonging and identity. Users who strongly identify with TMP not only continuously obtain tourism information on the platform, but are more intention to spend time and energy providing tourism information.

### Theoretical implications

Firstly, we theoretically enriched the research of users’ continued use intention, which divided into continuously obtain information and continuously provide information. Unlike previous research that have mainly used an overall uni-dimensional construct of users’ continued use intention (Pan et al., 2016; Choi et al., 2019; Lei and Zheng, 2021). This work also explored how users are driven to user continued use intention by integrating user experience with platform attachment, which contributes to the prior literature such as user experience (Lei and Zheng, 2021; Palos-sanchez et al., 2021) or platform attachment (Li et al., 2021) on user continued use intention.

Secondly, this work extends innovatively the SOR framework to the TMP context, unlike previous studies have mainly focus on in the field of e-commerce live streaming (Xu et al., 2020) and health care (Chen and Yin, 2021), and further deepens the understanding of continued use intention from the perspective of user experience. Specifically, the SOR framework explains how environmental stimuli (user experience) affect internal psychology (platform attachment) and thus result in behavioral outcomes (continued use intention). That is, the framework provides an overall view of the antecedents and consequences of platform attachment, which is helpful for a detailed understanding of the potential drivers of continued use intention.

Thirdly, this study filled the gap of user continued use intention research. Unlike prior studies that have mainly used an overall uni-dimensional construct of platform attachment (Li et al., 2021), this study innovatively divided platform attachment into platform dependence and platform identity, and found that platform identity is the most important driver of user continued use intention. Additionally, platform dependence will also positively affect users’ intention to continue using. The results of empirical analysis showed that the research framework can effectively explain and predict user continued use intention. Also, the data in this work reinforce the argument that an overall explanation of platform attachment’s effects is somewhat rough and misleading (Li et al., 2021). Platform dependence and platform identity have different psychological characteristics (Kyle et al., 2004), and thus exert different effects on the user continued used intention.

### Practical implications

This study also provides some practical implications. In order to promote users’ continued use intention, TMP operators should pay attention to the users’ functional experience and social experience with the TMP at the same time. For platforms, they can deeply cultivate and innovate basic products or services and improve organizational strategies, which can attract a large number of users (Saxton and Wang, 2016), and enhance communication between users. TMP operators should take some strategic actions to strengthen the users’ attachment to the TMP, which can help to retain users and promote more transactions.

Firstly, TMP operators realize the high-quality development of the platform from the aspects of hard technology and soft services, such as ensuring the reliability of tourism destination information, convenient reservation, regularly updating tourism products or services, protecting users’ privacy information, etc. The platforms should make full use of these measures to enhance users’ continued use intention.

Secondly, in the context of post-modern tourism highlighting personalized and emotional needs, managers of TMP need to build a tourism community to provide opportunities for users to communicate with each other, deepen the TMP social experience, and establish a solid relationship with the platform through the social interaction between users. For example, The way of game element is adopted (Ye et al., 2021), such as awarding medals and bonus points to users, which may encourage users to communicate with each other, meet social needs and cultivate platform recognition.

### Limitations and future research

This study has some limitations. First, all respondents are required to have used one TMP (i.e., Ctrip). Although Ctrip is a main TMP in China, there may be some differences between different TMPs (e.g., Mafeng and Qunar). Future studies should further test the results gained in this study in other TMP contexts. Second, there are certain limitations in the questionnaire survey. The continued use intention does not represent the actual behavior. Future research can collect actual using data of the platform, and analyze the actual causal relationship by adopting experimental and econometric designs. Third, this work attempts to incorporate relevant factors into the research model, but it is impossible to exclude other variables such as usage habits and familiarity that may also affect the dependent variable. Therefore, investigations of more holistic and more explanatory models of TMP user’s continued use intention are warranted in future studies.

## Data availability statement

The original contributions presented in the study are included in the article/supplementary material, further inquiries can be directed to the corresponding authors.

## Author contributions

GX is mainly responsible for the direction of the article. QC mainly wrote articles and questionnaires. QL is mainly responsible for the grasp of article logic, English translation, and data statistics. All authors contributed to the article and approved the submitted version.

## Funding

This work was supported by National Natural Science Foundation, China (No. 71772161 and No. 72172142) and Natural Science Foundation of Zhejiang Province, China (No. LQ20G020001).

## Conflict of interest

The authors declare that the research was conducted in the absence of any commercial or financial relationships that could be construed as a potential conflict of interest.

## Publisher’s note

All claims expressed in this article are solely those of the authors and do not necessarily represent those of their affiliated organizations, or those of the publisher, the editors and the reviewers. Any product that may be evaluated in this article, or claim that may be made by its manufacturer, is not guaranteed or endorsed by the publisher.
